# Survival of primary, but not of cancer cells after combined Plk1-HDAC inhibition

**DOI:** 10.18632/oncotarget.4445

**Published:** 2015-06-26

**Authors:** Lisa Lange, Peter Hemmerich, Birgit Spänkuch

**Affiliations:** ^1^ Friedrich-Schiller-University, CMB, Institute for Biochemistry and Biophysics, 07745 Jena, Germany; ^2^ Leibniz-Institute for Age Research-Fritz Lipmann Institute, JenAge (Jena Centre for Systems Biology of Aging), 07745 Jena, Germany

**Keywords:** polo-like kinase 1, HDAC, p21, cell cycle, cancer

## Abstract

In the current study we examined the combination of SAHA and SBE13 in cancer and non-cancer cells. HeLa cells displayed a synergistically reduced cell proliferation, which was much weaker in hTERT-RPE1 or NIH-3T3 cells. Cell cycle distribution differed in HeLa, hTERT-RPE1 and NIH-3T3 cells. SAHA-treated HeLa cells showed slightly increasing cell numbers in G2/M phase, but after combination with SBE13 strongly elevated cell numbers in G2/M and S phase, accompanied by decreasing G0/G1 percentages. hTERT-RPE1 and NIH-3T3 cells showed strongly enriched cell numbers in G0/G1 phase. Western blot and quantitative real time analyses revealed reduced Plk1 mRNA and protein in all cells. p21 protein was strongly induced in cancer, but not in non-cancer cells, corresponding to a different localization in immunofluorescence studies. Additionally, these revealed an abundantly present pRb protein in HeLa cells after any treatment but almost completely vanished pRb staining in treated hTERT-RPE1 cells. These differences could be approved in Western blots against Parp and Caspase 3, which were activated in HeLa, but not in hTERT-RPE1 cells. Thus, we observed for the first time a differential effect of cancer versus non-cancer cells after treatment with SAHA and SBE13, which might be due to the dual role of p21.

## INTRODUCTION

The search for new therapeutic strategies is an important research field in translational cancer research. Many strategies inhibit one or more cancer-relevant genes with the aim to inhibit cancer cell proliferation by inhibiting the expression or activity of the targeted genes. One promising strategy to improve therapy is the use of small molecule kinase inhibitors, which represent essential tools in basic and translational research.

One attractive target gene in the fields of signaling research and cancer therapy is the serine/threonine kinase Plk1 (polo-like kinase 1) [1], which shows elevated activity in all human tumors [2–4]. Plk1 plays a pivotal role for mitosis and as a measure for the aggressiveness of a tumor due to its important role for the mitotic checkpoints of cancer cells [5–10]. Plk1 has predictive and prognostic value for cancer patients [10, 11]. Interfering with Plk1 activity and/or expression using dominant-negative mutants, antibody microinjection, antisense oligonucleotides, small interfering RNAs or kinase inhibitors leads to different mistakes in centrosomal maturation, mitotic catastrophe and increased apoptosis in cancer cells [8, 11–21]. In addition to its role during mitosis, Plk1 has multiple functions outside of mitosis, for example for checkpoint recovery after DNA damage [11, 22] and for G1/S phase [23].

SBE13, a selective type II Plk1 inhibitor, is able to induce a delay in cell cycle progression, to reduce cell proliferation and to induce apoptosis in a broad range of human cancer cell lines [16, 17]. SBE13 displayed 1,000-fold selectivity towards Plk family members and did not influence Aurora A activity. SBE13 displayed a differential effect between cancer and primary cells [24] with especially G2/M arrest in cancer cells and transient G0/G1 arrest in primary cells without any signs of cell death, confirming earlier studies [18, 21].

Histone deacetylases (HDAC) suppress transcription of their specific target genes and are correlated to cancer pathogenesis. They counteract the activity of histone acetyl transferases, which acetylate for example histones to make them accessible for transcription factors. These acetylations are removed by HDACs afterwards. Additionally, it has been described that HDAC inhibitors (HDACi) reduce Plk1 protein, suggesting the combination of Plk1 and HDAC inhibitors to combat cancer [25, 26].

Most of the analyzed tumor cells respond to an inhibition of HDAC with signs of apoptosis, growth/proliferation arrest, differentiation and altered gene expression. However, in many cases it is unclear whether these effects resulted from an altered gene-expression profile or were caused by toxic effects inherent to the substances employed [27]. Some of them are already tested in clinical studies [28]. Various studies describe an induction of apoptosis by HDACi which is a consequence of non-functional cell cycle checkpoints [29, 30]. Interestingly, the reduction of the Cdk inhibitor p21^WAF/CIP1^, which is responsible for G1 arrest of cells, can induce HDACi-induced cell death [31, 32] although p21 is upregulated by HDACi [33].

In this article, we report the combination of SBE13 with the HDACi SAHA (suberanilohydroxamic acid, Vorinostat) in cancer (HeLa cells) and non-cancer cells (NIH-3T3 and hTERT-RPE1 cells). Potential differential effects were elucidated by the analysis of Plk1 mRNA and protein, the induction of apoptosis, the reduction of proliferation, of pRb and p21 levels, of protein expression of target proteins, and of the cell cycle distribution.

## RESULTS

### Reduced Plk1 mRNA after treatment with SAHA and with SAHA and SBE13 together in HeLa, hTERT-RPE1 and NIH-3T3 cells

**Figure 1:**
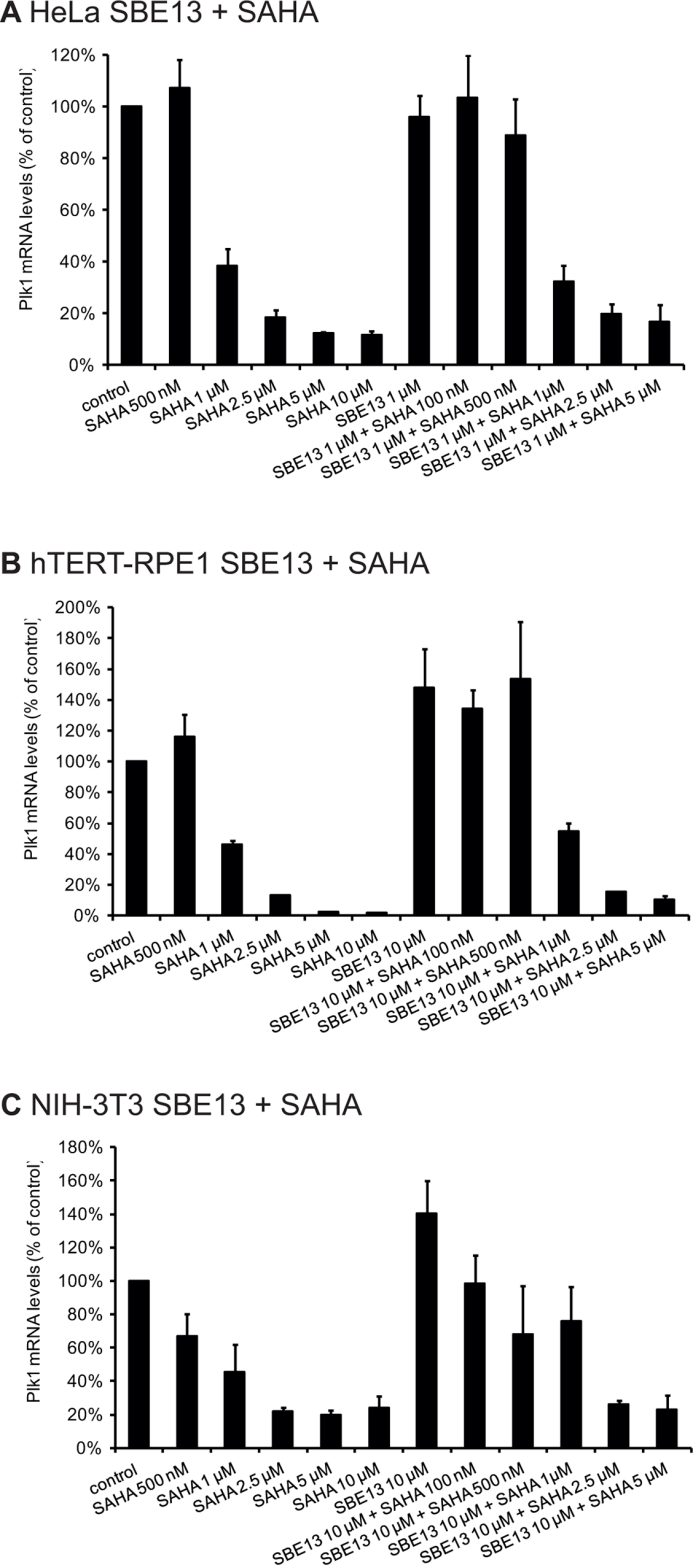
Quantitative real-time analysis of HeLa, hTERT-RPE1 and NIH-3T3 cells after incubation with SAHA and SBE13 using Plk1- and GAPDH-specific primers Quantitative real-time analysis of Plk1 mRNA levels 24 hrs after treatment with SAHA alone and in combination with SBE13 in HeLa **A.** hTERT-RPE1 **B.** and in NIH-3T3 cells **C.** Graphical summary of gene expression values of treated cells standardized to control cells are shown (*n* = 3 × 3, mean ± SD).

First, we determined the levels of Plk1 mRNA after treatment with SAHA alone and in combination with SBE13 in HeLa (cancer cells), hTERT-RPE1 (non-transformed immortalized) and NIH-3T3 cells (mouse fibroblasts) (Figure 1). In HeLa cells, we observed statistically significantly reduced mRNA levels after single and combinatorial treatment with 1 to 10 μM SAHA (1 μM: 39%, *p* = 0.008, 2.5 μM: 18%, *p* = 0.0004, 5 μM: 12%, *p* < 0.0001, 10 μM: 12%, *p* < 0.0001) alone and with 1 to 5 μM SAHA in combination with 1 μM SBE13 (1 μM: 32%, *p* = 0.002, 2.5 μM: 20%, *p* = 0.0007, 5 μM: 17%, *p* = 0.002) (Figure 1A). In hTERT-RPE1 cells effects were comparable with reductions to 46% with 1 μM SAHA (*p* = 0.0007), to 13% with 2.5 μM SAHA (*p* < 0.0001), to 2% with 5 μM SAHA (*p* < 0.0001), and to 2% with 10 μM SAHA (*p* <0.0001) (Figure 1B). In combination with 10 μM SBE13 effects were comparable to SAHA alone showing reductions to 55% with 1 μM SAHA (*p* = 0.005), to 15% with 2.5 μM SAHA (*p* < 0.0001), and to 10% with 5 μM SAHA (*p* = 0.0002). In NIH-3T3 cells similar effects could be observed (Figure 1C): reduction to 46% with 1 μM SAHA (*p* = 0.028), to 22% with 2.5 μM SAHA (*p* = 0.0004), to 20% with 5 μM SAHA (*p* = 0.0003), and to 24% with 10 μM SAHA (*p* = 0.002). As in HeLa and in hTERT-RPE1 cells the reduction of Plk1 mRNA was not stronger, but even less pronounced after combinatorial treatment with SBE13 (10 μM SBE13: reduction to 26% with 2.5 μM SAHA (*p* = 0.0002), and to 23% with 5 μM SAHA (*p* = 0.004). These effects suggest an interference of HDAC inhibitors with transcriptional regulation of Plk1 in cancer and in non-cancer cells which is—as expected—not influenced by additional inhibition of Plk1 activity.

### Reduced levels of Plk1 protein after treatment with SAHA and with SAHA and SBE13 together in HeLa, hTERT-RPE1 and NIH-3T3 cells

**Figure 2:**
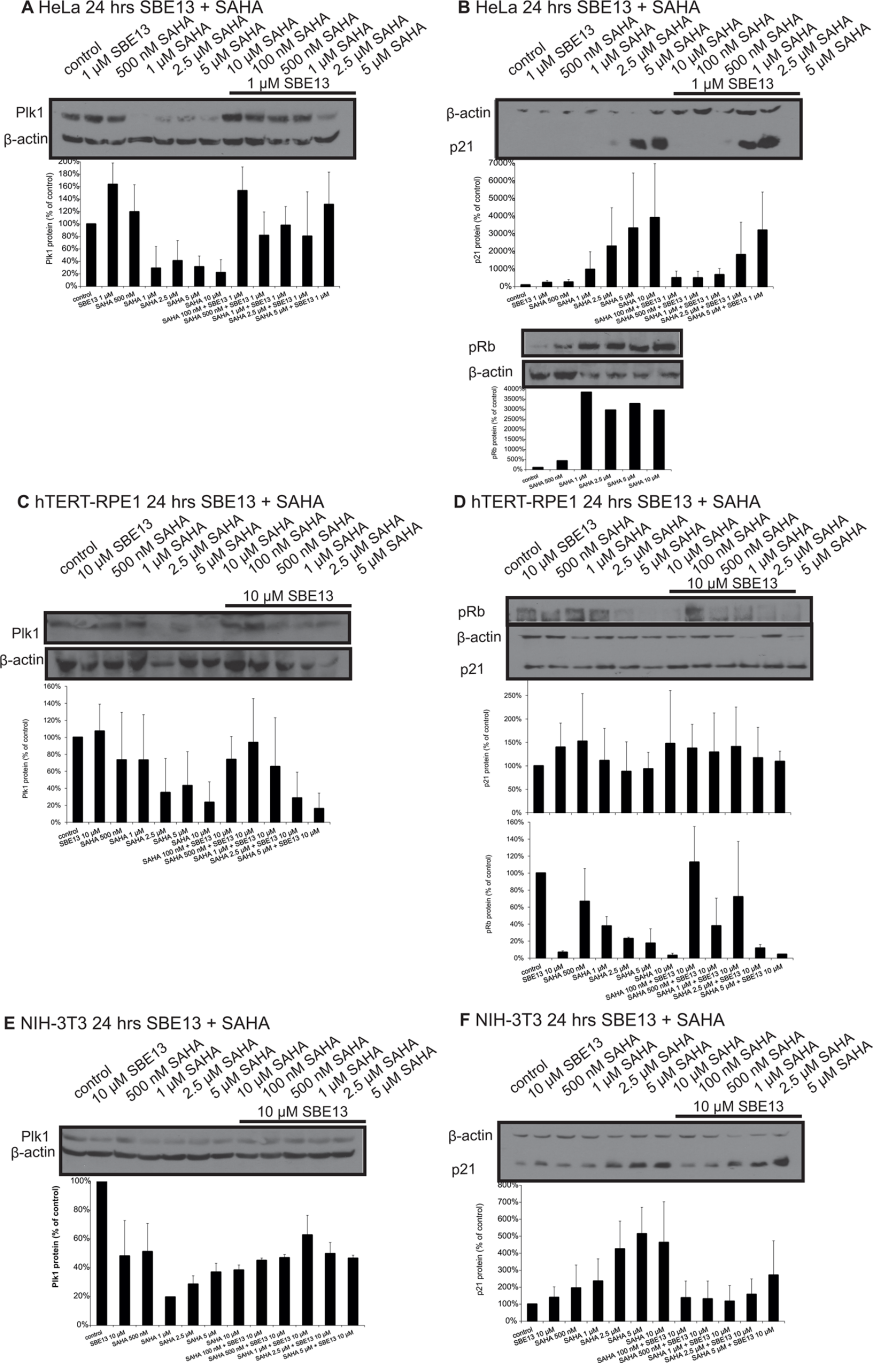
Western Blot analyses of Plk1 and p21 protein expression and of pRb levels in HeLa, hTERT-RPE1 and NIH-3T3 cells after treatment with SAHA and SBE13 Western blot analysis of Plk1 protein expression in HeLa **A.** hTERT-RPE1 **C.** and NIH-3T3 cells **E.** 24 hrs after treatment with SAHA alone and in combination with SBE13 (HeLa cells 1 μM SBE13, hTERT-RPE1 and NIH-3T3 cells 10 μM). Western blot analysis of p21 protein expression and pRb levels in HeLa **B.** hTERT-RPE1 **D.** and p21 protein expression in NIH-3T3 cells **F.** 24 hrs after treatment with SAHA alone and in combination with SBE13 (HeLa cells 1 μM SBE13, hTERT-RPE1 and NIH-3T3 cells 10 μM). Figures show representative blots and graphical summary.

To analyze whether the reduction of Plk1 mRNA resulted in decreased protein levels we did Western blot analyses targeting Plk1 in HeLa, hTERT-RPE1 and NIH-3T3 cells (Figure 2A, 2C, 2E). In all three cell lines, regardless whether they are cancer cells (HeLa), non-transformed immortalized cells (hTERT-RPE1) or completely normal fibroblasts (NIH-3T3) the Plk1 protein was significantly reduced by SAHA treatment. We observed reductions to levels between 4 and 38% with 1 to 10 μM SAHA alone in HeLa cells, which were less pronounced in combination with 1 μM SBE13 (levels of 48–60%, Figure 2A). In hTERT-RPE1 cells Plk1 protein was reduced to levels between 23 and 73% with 500 nM–10 μM SAHA, and to levels of 16 to 74% with 10 μM SBE13 in combination with 100 nM–5 μM SAHA (Figure 2C). Comparable effects could be observed in NIH-3T3 cells, where we detected reductions of Plk1 protein levels to 20 to 51% with 500 nM – 10 μM SAHA alone, and in combination with 10 μM SBE13 Plk1 protein levels were reduced to levels of 45–63% with SAHA concentrations from 100 nM to 5 μM (Figure 2E).

### Different regulation of p21 and pRb after treatment with SAHA and SBE13 in HeLa, hTERT-RPE1 and NIH-3T3 cells

To further investigate the underlying mechanism we did Western blot analyses in HeLa, hTERT-RPE1 and NIH-3T3 cells targeting p21 (Figure 2B, 2D, 2F). We observed a strong induction of p21 protein in HeLa cells after single and combinatorial treatment to levels of nearly 4000% (Figure 2B), which could not be observed in hTERT-RPE1 cells, which showed p21 levels between 100 and 150% compared to control cells (Figure 2D). NIH-3T3 cells showed an increase of p21 protein, but this induction was much weaker than in HeLa cells (a maximum induction to approx. 500% compared to almost 4000% in HeLa cells, Figure 2F).

Additionally a cell cycle marker, phosphorylated Rb protein, was analyzed to distinguish between cell cycle arrest types or apoptosis induction in HeLa and hTERT-RPE1 cells. hTERT-RPE1 cells showed the expected strong decreased phospho-Rb protein after SBE13 treatment which has been already described [24], but in addition, we could see a strong decrease after SAHA or the combinatorial treatment (Figure 2D, lower panel). In contrast, HeLa cells showed a strong increase of phospho-Rb after SAHA treatment (Figure 2B, lower panel) corresponding to an override of the G1/S checkpoint as observed in earlier studies also for SBE13 [24].

### The proteasome inhibitor MG132 does not abrogate the reduction of Plk1 or p21 protein after treatment with SAHA in HeLa cells

**Figure 3:**
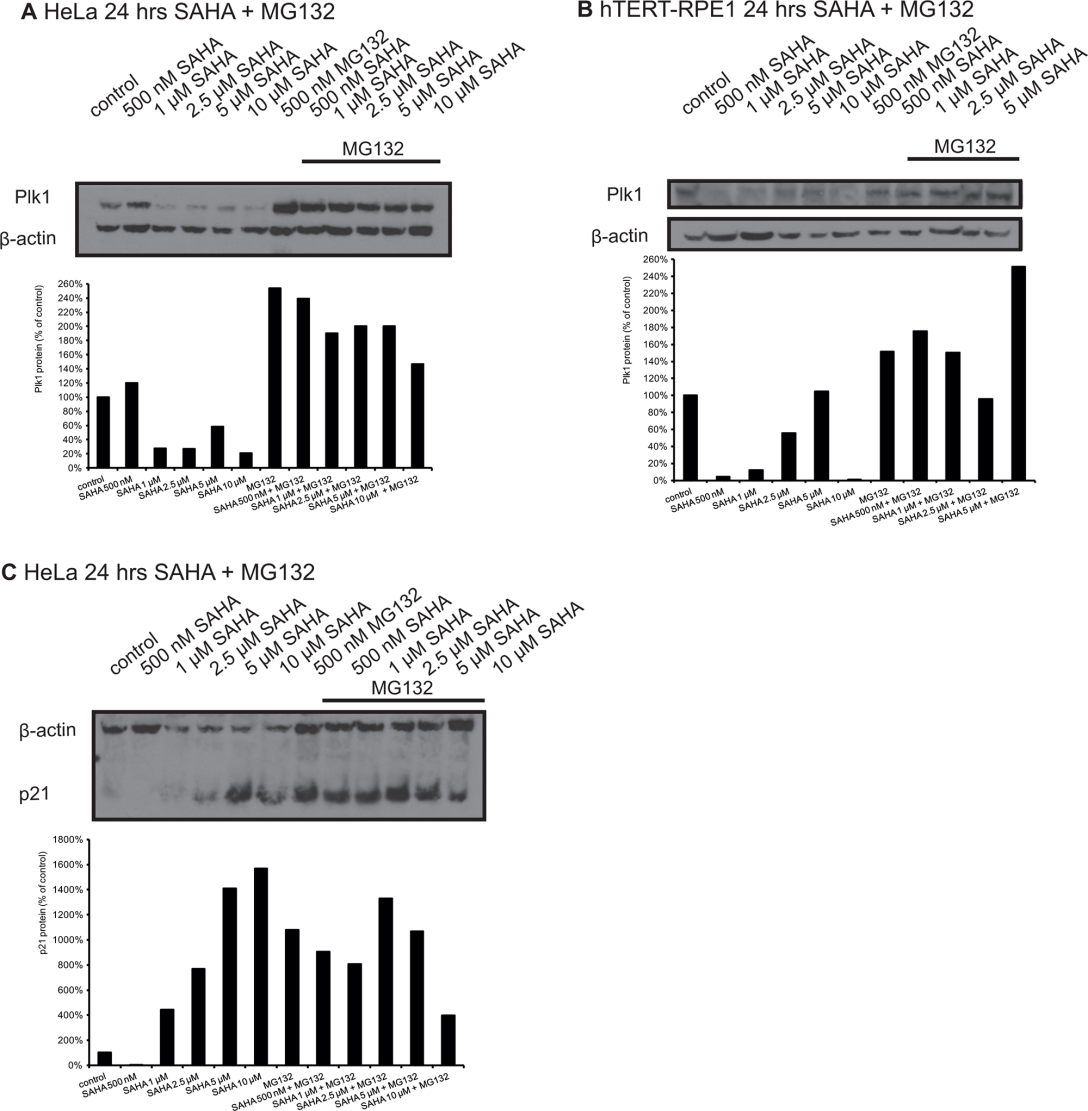
Western Blot analyses of Plk1 protein expression in HeLa and hTERT-RPE1 cells after treatment with SAHA and MG132 Western blot analysis of Plk1 protein expression in HeLa **A.** and hTERT-RPE1 cells **B.** 24 hrs after treatment with SAHA and MG132. **C.** p21 levels in HeLa cells 24 hrs after treatment with SAHA and MG132. Figures show representative blots and graphical summary.

To confirm the real-time PCR data concerning reduced Plk1 mRNA by SAHA treatment and thus to confirm the proposed involvement of transcriptional regulation of Plk1 by HDAC inhibitors, we did Western blot analyses after incubation of HeLa and hTERT-RPE1 cells with SAHA together with the proteasome inhibitor MG132 (Figure 3A, 3B). We observed in both cell types that SAHA reduced Plk1 protein levels very strongly, and that MG132 inhibits the proteasomal degradation of Plk1 demonstrated by strong enrichment after MG132 treatment. But the combination of SAHA with MG132 reduced Plk1 protein compared to MG132 treatment alone, which is a further hint that the reduction of Plk1 protein after SAHA treatment is due to the observed regulation on Plk1 transcription by SAHA and not due to enhanced protein stability.

In addition, we analyzed p21 protein levels after SAHA treatment in combination with MG132 in HeLa cells, and it is obvious that MG132 inhibits the proteasomal degradation of p21, but it is only slightly induced after SAHA treatment with MG132 compared to MG132 alone (Figure 3C).

### Cell cycle analysis of HeLa, hTERT-RPE1 and NIH-3T3 cells after treatment with SAHA and SBE13

We did FACS analyses to determine the cell cycle distribution of HeLa, hTERT-RPE1, and NIH-3T3 cells to examine whether the changes in Plk1 and HDAC activity were associated with an arrest in particular stages of the cell cycle and if these cell cycle arrests are time-dependent. In addition, we wanted to figure out whether the absence or presence of a functional G1/S checkpoint influences the cell cycle arrest.

**Figure 4:**
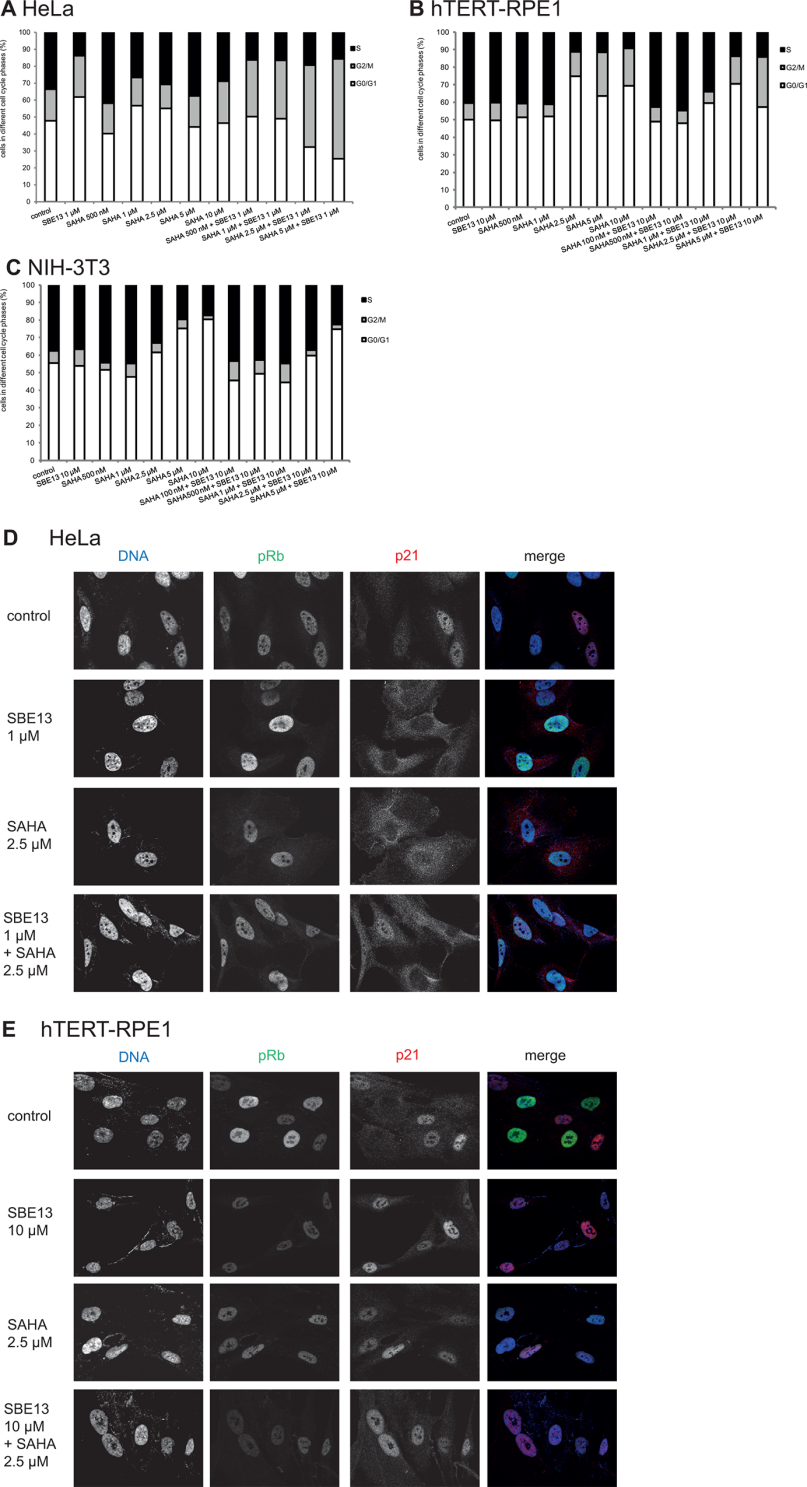
Effect of SAHA and SBE13 on cell cycle distribution of HeLa A. hTERT-RPE1 B. and NIH-3T3 cells C. and immunofluorescence microscopy of HeLa D. and hTERT-RPE1 cells E (A–C): Cells were incubated for 48 hours with SAHA alone and in combination with SBE13 and analyzed for their cell cycle distribution. The graphs show the relative number of cells in the respective cell cycle phases. (D, E): Cells were incubated for 24 hrs with SAHA alone and in combination with SBE13 and analyzed for phosphorylated Rb and for p21 protein using CLM microscopy. Red: p21, green: phospho-Rb, blue: DNA staining. Acquisition settings (exposure times) were kept constant for all images and were as follows: 200 ms for DAPI, 1 s for Alexa 488 and 500 ms for Cy3. Optical sections with an axial resolution of 720 nm (DAPI), 790 nm (Alexa488) and 880 nm (Cy3) were achieved through structured illumination employing 3 phase images without any normalization, phase correction, Fourier filtering or deconvolution.

First, we analyzed HeLa cells and observed an increase of cells in G2/M phase after treatment with SAHA alone and a very prominent increase of cells in G2/M phase and in S phase while G0/G1 phase is strongly reduced after the combination of SAHA with SBE13 (Figure 4A). In hTERT-RPE1 cells, we observed a different cell cycle distribution (Figure 4B): SAHA alone and SAHA together with SBE13 differed only slightly, both inducing a slight increase of cells in G2/M phase and a very strong enrichment of cells in G0/G1 phase. NIH-3T3 cells showed strongly elevated numbers of cells in G0/G1 phase after single and combinatorial treatment (Figure 4C), which is in concordance with the expectation based on our own studies using SBE13 in primary cells [24].

### Immunofluorescence analysis of pRb and p21 after treatment with SAHA and SBE13 in HeLa and hTERT-RPE1 cells

To analyze the differences between HeLa and hTERT-RPE1 cells in more detail we did immunofluorescence analyses in SBE13- and SAHA-treated cancer and non-cancer cells (Figure 4D, 4E). The phospho-Rb protein is indicative of a functional G1/S checkpoint, and our own studies using SBE13 revealed an override of G1/S checkpoint in HeLa cells and a G1/S checkpoint arrest in hTERT-RPE1 cells [24]. To analyze whether this difference in pRb is also the reason for the different response of HeLa and hTERT-RPE1 cells after SBE13 and SAHA treatment we analyzed pRb protein in both cell types. HeLa cells showed pRb staining in all control cells with some variation in signal intensity in individual nuclei (Figure 4D, upper panel). After SBE13, SAHA and the combinatorial treatment the pRb staining pattern stayed essentially the same (Figure 4D, lower three panels). In striking contrast, hTERT-RPE1 control cells displayed many nuclei showing strong staining, and some cells with very low pRb levels (Figure 4E, upper panel). After treatment with SBE13 or SAHA alone and after the combinatorial treatment pRb staining was strongly diminished in all nuclei (Figure 4E, lower three panels). These observations suggest a functional p21/pRb-mediated G1/S checkpoint in hTERT-RPE1 as a cause for the differences in cell proliferation and apoptosis induction (see below) after the treatments in comparison to HeLa cells.

In addition, we analyzed p21 protein to investigate potential differences in localization or induction/reduction in HeLa and hTERT-RPE1 cells (Figure 4D, 4E). We detected p21 protein in HeLa cells mainly within the nuclei (Figure 4D, upper panel). In contrast, after SBE13 treatment p21 was localized preferentially within the cytoplasm (Figure 4D, second panel). After SAHA treatment, alone or in combination with SBE13, we detected p21 throughout the whole cell without the strong cytoplasmic enrichment as observed for SBE13 alone. hTERT-RPE1 cells did not show this treatment-induced differential p21 (re)localization but was predominantly detectable in the nuclei of these immortalized cells (Figure 4E).

### Analysis of apoptosis induction after treatment with SAHA and SBE13 in HeLa and hTERT-RPE1 cells

**Figure 5:**
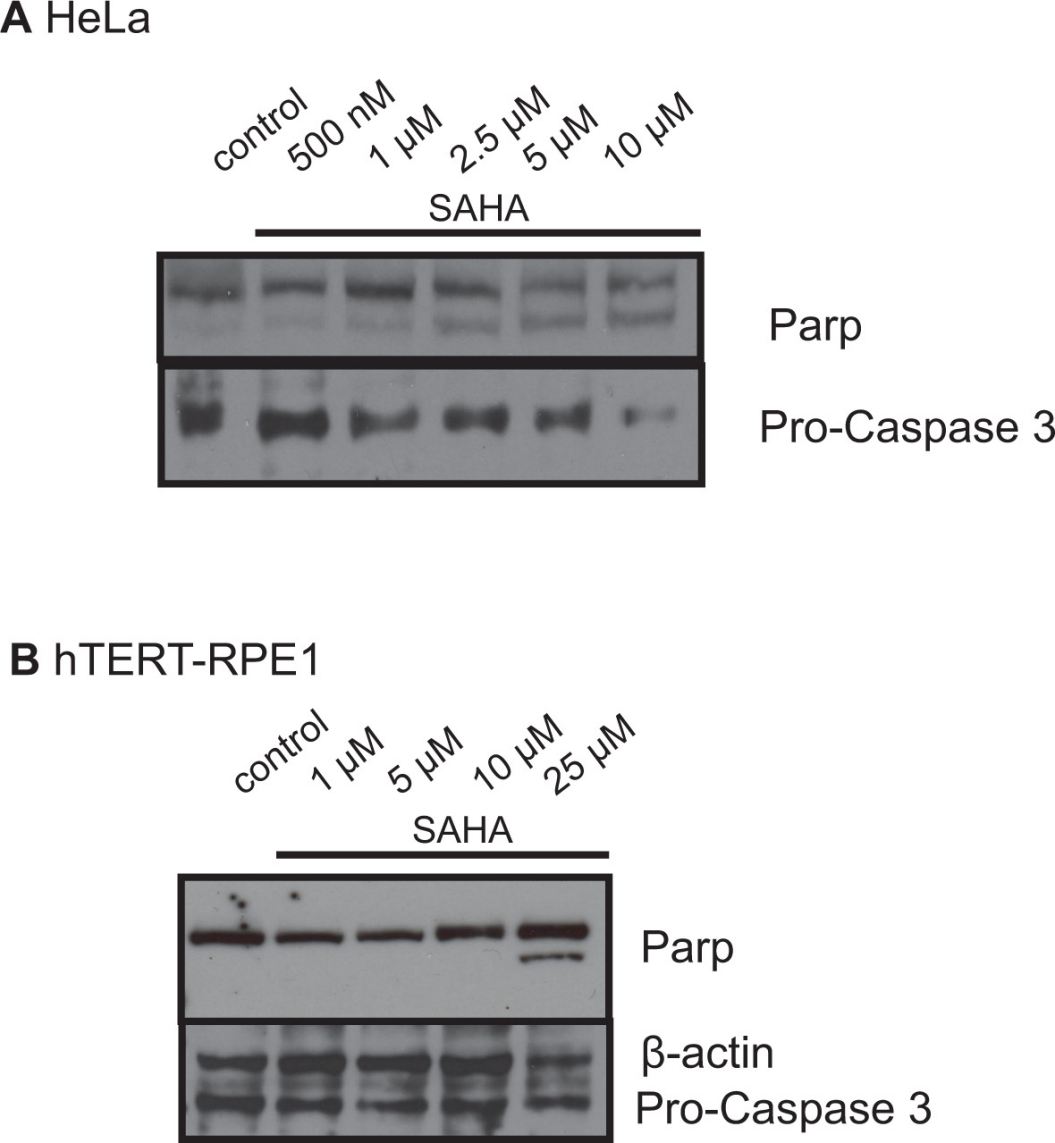
Western blot analyses of Parp and Caspase 3 in HeLa A. and hTERT-RPE1 cells B. after treatment with SAHA Figures show representative blots after incubation with antibodies against Parp and Pro-Caspase 3.

We did Western blot analyses targeting Parp and Caspase 3 after SAHA treatment of HeLa and hTERT-RPE1 cells to determine potentially different reactions of cancer and non-cancer cells to SAHA treatment. We observed strikingly differing induction of Parp cleavage and Pro-Caspase 3 activation (Figure 5): In HeLa cells an increasing Parp cleavage could already be detected after treatment with 1 μM SAHA (Figure 5A), while hTERT-RPE1 cells did not show any Parp cleavage up to 10 μM SAHA, but only an increase of the cleavage product after treatment with 25 μM SAHA (Figure 5B). The same observation could be made for activation of Pro-Caspase 3 with almost no induction in hTERT-RPE1 cells, while in HeLa cells Pro-Caspase 3 was already activated after treatment with 1 μM SAHA according to the Parp cleavage.

### Cell proliferation analysis after treatment with SAHA and SBE13 in HeLa, hTERT-RPE1 and NIH-3T3 cells

**Figure 6:**
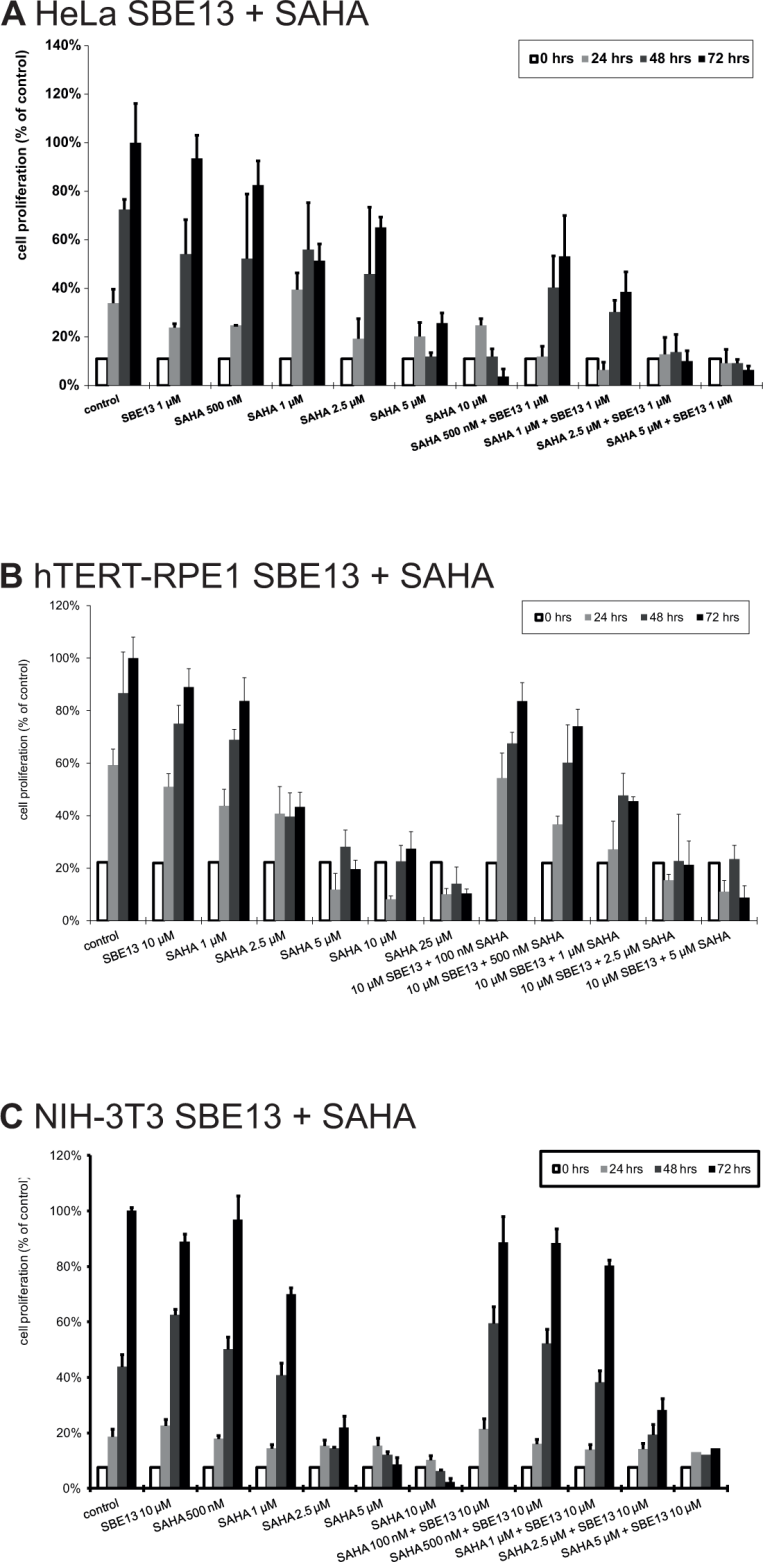
Cell proliferation of HeLa A. hTERT-RPE1 B. and NIH-3T3 cells C. after treatment with SAHA and SBE13 Cells were incubated for 24–72 hours with SAHA alone or in combination with SBE13 (HeLa cells: 1 μM, hTERT-RPE1 and NIH-3T3 cells: 10 μM). Percentage of surviving cells is given as percentage of the number of control cells after 72 hrs. Bar graphs represent means of three different experiments.

To investigate whether the cell cycle arrest and induction of apoptosis causes a reduction of cell proliferation, we analyzed the cell proliferation of the various cell lines after treatment with SAHA alone and in combination with SBE13 (Figure 6).

HeLa cells showed a slightly reduced cell proliferation to levels of 83% with 500 nM SAHA and to 51% with 1 μM SAHA (Figure 6A). The reduction was significant with 2.5 μM SAHA (65%, *p* = 0.04) and with 5 and 10 μM SAHA (26%, *p* = 0.02 and 4%, *p* = 0.008, respectively) (Figure 6A). In combination with 1 μM SBE13 we observed a significant reduction already with 500 nM SAHA (53%, *p* = 0.01). Together with 1 μM SAHA to 39%, *p* = 0.01, with 2.5 μM SAHA to 10%, *p* = 0.008, and with 5 μM SAHA to 6%, *p* = 0.008. This stronger reduction in combination was synergistic with a c.i. value of 0.21.

In contrast, hTERT-RPE1 and NIH-3T3 cells displayed a weaker combinatorial effect on the reduction of cell proliferation. hTERT-RPE1 cell proliferation was reduced to 84% with 1 μM SAHA (*p* = 0.16), to 43% with 2.5 μM SAHA (*p* = 0.003), to 20% with 5 μM SAHA (*p* = 0.004), to 27% with 10 μM SAHA (*p* = 0.001) and to 10% with 10 μM SAHA (*p* = 0.004) (Figure 6B). The combination with SBE13 showed a slightly stronger effect on cell proliferation (10 μM SBE13 + 100 nM SAHA reduction to 84%, *p* = 0.06, SBE13 + 500 nM SAHA reduction to 74%, *p* = 0.08, SBE13 + 1 μM SAHA reduction to 45%, *p* = 0.007, SBE13 + 2.5 μM SAHA reduction to 21%, *p* = 0.003, and SBE13 + 5 μM SAHA reduction to 9%, *p* = 0.001). This combination was synergistic with a c.i. value of 0.5, thus much less than in the HeLa cancer cells. NIH-3T3 cells showed already a very strong reduction of cell proliferation after single SAHA administration (Figure 6C) with a reduction to 70% with 1 μM, *p* = 0.003, to 22% with 2.5 μM, *p* < 0.001, to 9% with 5 μM, *p* < 0.001, and to 2% with 10 μM SAHA, *p* < 0.001. The combination with 10 μM SBE13 led to slightly stronger reduced cell proliferation: together with 100 or 500 nM SAHA, respectively, proliferation was not reduced compared to control cells (reduction to 89%, *p* = 0.138 or 0.07, respectively), with 1 μM SAHA to 80%, *p* = 0.003, with 2.5 μM to 28%, *p* < 0.001, and with 5 μM SAHA 14%, *p* = 0.001. This reduction of cell proliferation was even not synergistic, but antagonistic (c.i. = 1.23).

## DISCUSSION

In the current study we analyzed for the first time the effects of SAHA in combination with SBE13 on cell cycle regulation, Plk1 expression, cell proliferation, and induction of apoptosis in HeLa cells, hTERT-RPE1 cells and in mouse fibroblast NIH-3T3 cells. From earlier studies it is known that Plk1 depletion leads to different effects in cancer vs. non-cancer cells, and thus we analyzed this combination in cancer and non-cancer cells to confirm these differential effects [18, 21, 24, 34].

First we wanted to point out whether SAHA influences Plk1 transcription or protein stability. Our quantitative real-time PCR and Western blot analyses revealed a regulation of Plk1 at the transcriptional level by SAHA, because in cancer and non-cancer cells Plk1 mRNA levels were statistically significantly reduced by SAHA (alone and in combination with SBE13). As expected, the combination of SAHA with SBE13 did not alter Plk1 transcript levels compared to SAHA alone. To rule out an influence of increased proteasomal degradation we did Western blot analyses using MG132-treated cells, but also after addition of MG132 Plk1 levels decreased following SAHA treatment compared to controls treated with MG132 alone. This observation is in concordance with the observation of Noh et al. regarding a down-regulation of Plk1 transcripts by Trichostatin A [25]. Indeed, Plk1 is a target of the Rb pathway via SWI/SNF-dependent histone deacetylation of the Plk1 promoter which can explain these effects [35].

This fact motivated us to analyze pRb and p21. Our Western blot analyses revealed a differential effect between cancer and non-cancer cells concerning p21 induction and apoptosis induction. HeLa cells showed a strong induction of p21 protein expression and Parp cleavage as well as Caspase 3 activation, while hTERT-RPE1 cells showed no p21 or apoptosis induction. In contrast to cancer cells non-transformed cells have higher basal levels of p21 as it is known that lacking of p21 leads to tumorigenesis [36]. Taking into account that we observed in earlier studies using SBE13 in HeLa cells a G2/M arrest and in hTERT-RPE1 cells a transient G0/G1 arrest [24], we assume that the differences between HeLa and hTERT-RPE1 are due to a functional G1/S checkpoint in hTERT-RPE1 cells and to the function of p21 as well at the G1/S and the G2/M transition [37, 38]. Another explanation for the different effects in cancer and non-cancer cells could be the localization of p21: it is well-known that cytoplasmic localization of p21 leads to an inhibition of Caspase 3 activation and thus to reduced apoptosis induction, and that p21 has a dual role as oncogene or tumor suppressor gene dependent on its localization [37, 39, 40]. If p21 is localized in the nucleus it works as a tumor suppressor, and in the cytoplasm as an oncoprotein [40]. We detected p21 in hTERT-RPE1 cells exclusively in the nuclei, supporting its role as tumor suppressor especially in cells with functional checkpoints. Due to this localization p21 can contribute to DNA repair, induction of cell cycle arrest and work as transcriptional cofactor [40]. In contrast, in HeLa cells we found a stronger cytoplasmic localization after treatment supporting its role as an oncoprotein and confirming earlier studies from other groups showing cytoplasmic enrichment of p21 after Plk1 inhibition [40] and thus leading to a prevention of apoptosis induction in cancer cells [39, 41]. After addition of SAHA or SAHA together with SBE13 p21 was found within the whole cell, losing its ability to prevent cells from apoptosis.

The fact that HeLa cells induce p21 after treatment with an HDAC inhibitor and hTERT-RPE1 cells do not, in combination with the observation that HeLa cells induce apoptosis while hTERT-RPE1 cells do not, is in concordance with Papeleu et al., who report that hepatoma cells show an upregulation of p21, accompanied by increasing Caspase 3 activity and subsequent apoptosis induction, while primary hepatocytes do not show this p21 induction and they do not show an activation of Caspase 3 and no signs of cell death [42]. Another publication corroborates the observation that NIH-3T3 cells show a slight induction of p21 after DNA damage, although these are non-cancer cells, because it is described that this increase can occur also in normal untransformed cells [37].

Additionally, it is known that p21 prevents Plk1 expression via direct binding of the Plk1 promoter, which may be part of an adaptive stress response [43]. Thus, generally, p21 contributes to G1 phase arrest via inhibition of Cyclin E, Cyclin A/Cdk activity and thus leaving Rb hypo-phosphorylated and sequestering E2F, whose activity is necessary for S phase entry [37]. This fits perfectly with our observation that hTERT-RPE1 cells show hypo-phosphorylated Rb protein after treatment with SBE13 and / or SAHA.

Our study is also consistent with observations identifying L-Carnitine as an endogenous HDACi, which selectively induced cytotoxic effects in cancer cells although it inhibited HDAC activity also in primary cells [44]. This confirms our observation that SAHA reduces Plk1 transcript and protein levels in both cancer and primary cells, but induces p21 and apoptosis, holds up phosphorylation of the Rb protein, leads to G2/M enrichment and to strongly reduced cell proliferation only in cancer cells, but not in non-cancer cells.

The current study provides another hint for the importance of an intact G1/S checkpoint in primary versus cancer cells for a differential effect in cancer therapy as observed in earlier studies [24]. Here, we could show for the first time, that the p21 protein localizes in hTERT-RPE1 cells mainly in the nucleus (in control and treated cells) confirming its role as a tumor suppressor gene [45]. In HeLa cells, in contrast, we observed many cells with cytoplasmic localization of p21, contributing to its known role as an oncoprotein, preventing apoptosis induction in HeLa cells in the absence of additional stresses. Addition of SAHA and SBE13 led to cell death of HeLa cells confirming the synergistic reduction of cell proliferation in contrast to non-cancer cells. Thus, p21 can execute its function as a tumor suppressor and G1/S regulator only in non-cancer cells, making it an interesting target for potential therapeutics.

## MATERIALS AND METHODS

### Kinase and HDAC inhibitors and antibodies

The Plk1 kinase inhibitor SBE13 was purchased from the SPECS compound catalogue (Delft, Netherlands), the HDAC inhibitor SAHA was from Selleck (Absource Diagnostics GmbH München, Germany).

Monoclonal-anti-Plk1, monoclonal-anti-Pro-Caspase 3, goat anti-mouse and goat anti-rabbit secondary antibodies were from Santa Cruz Biotechnology, Inc., (Heidelberg, Germany), rabbit anti-phospho-Rb antibody was from abcam (Cambridge, UK), monoclonal-anti-p21 and rabbit-anti-Parp antibodies were from Cell Signaling (Frankfurt/Main, Germany) and monoclonal β-actin-antibody was from Sigma-Aldrich (Taufkirchen, Germany). Monoclonal p21-antibody for immunofluorescence was from BD Biosciences (Heidelberg, Germany). Cy3-conjugated goat anti-rabbit IgG F(ab')2-fragment antibody was from Dianova (Hamburg, Germany) and Alexa488-conjugated goat-anti-mouse antibody was from Jackson ImmunoResearch Laboratories (Baltimore, USA).

### Cell culture

HeLa and NIH-3T3 cells were from DSMZ (Braunschweig, Germany), hTERT-RPE1 cells were from Clontech (Saint-Germain-en Laye, France). All cells were cultured according to the supplier's instructions without antibiotics. Fetal calf serum (FCS) was from PAA Laboratories (Cölbe, Germany), MEM, DMEM, DMEM F12, phosphate buffered saline (PBS), glutamine, and trypsin were from Invitrogen (Karlsruhe, Germany).

### Treatment and analysis of cells

Cells were treated with SBE13 and SAHA alone or in combination one day after subculturing. Cells were seeded onto 6-well-plates, or 75-cm^2^- flasks, respectively. Control cells were incubated with normal culture medium without antibiotics. Concentrations of SBE13 were 1 μM for cancer cells and 10 μM for non-cancer cells, respectively, SAHA concentrations ranged from 0.1–10 μM. The growth rate of 5 × 10^4^ cells per 6-well was determined by counting cells at 24 to 72 hours after treatment using a hematocytometer. Cell culture studies were performed in triplicate for each time point. Cells were harvested 24–72 hours after treatment for further analyses.

### Quantitative real-time PCR analysis

After isolation of total RNA using RNeasy mini-kits (Qiagen, Hilden, Germany) according to the manufacturer's protocol 24 hrs after incubation with SAHA alone or together with SBE13, respectively, the mRNA was transcribed into cDNA using the High-Capacity cDNA Reverse Transcription Kits (Applied Biosystems, Darmstadt, Germany). Thereafter, 50 ng cDNA were subjected to quantitative real-time PCR analyses targeting Plk1 and GAPDH using the FastStart Universal Probe Master ROX and Universal ProbeLibrary #30 (Plk1) and #60 (GAPDH) (Roche Applied Biosciences, Mannheim, Germany). Primer sequences were chosen as suggested by Roche Applied Biosciences (Mannheim, Germany): Plk1 forward 5′-cacagtgtcaatgcctccaa, Plk1 reverse 5′-ttgctgacccagaagatgg, GAPDH forward 5′-agccacatcgctcagacac, GAPDH reverse 5′-gcccaatacgaccaaatcc. Analysis was performed using the StepOne Plus RealTime PCR System and the StepOne v2.2.3 software (Applied Biosystems, Darmstadt, Germany). Relative gene expression values were determined by the ΔΔC_T_ method using the StepOne v2.3 software (Applied Biosystems, Darmstadt, Germany). Data are presented as the fold difference in Plk1 expression normalized to the housekeeping gene GAPDH as endogenous reference, and relative to the untreated control cells.

### Western blot analysis

Total protein (50 μg) was separated on 10% Bis-Tris-polyacrylamide gels and transferred (at 30 V for 1 hr) to Immobilon™-P membranes (Millipore, Bedford, MA) according to the Invitrogen protocol (Karlsruhe, Germany). Membranes were incubated for 1 hr in 5% powdered nonfat milk in PBS with antibodies against Plk1 (1:200), Pro-Caspase 3 (1:1,000), p21 (1:2,000), pRb (1:2,000), Parp (1:1,000) or β-actin (1:100,000) and for 30 min in 5% nonfat dry milk with goat anti-mouse or goat anti-rabbit serum (1:2,000) and visualized as described [21].

All protein expression levels were presented as described [21], scanned and quantified with the freeware ImageJ (National Institutes of Health, USA).

### FACS analysis

Cell cycle distribution was analyzed using a FACScalibur apparatus (Becton Dickinson, Heidelberg, Germany). Quantification was carried out using ModFit LT 3.3 for Windows (Verity Software House, Topsham, ME). For FACS analysis, cells were harvested at the indicated time points, washed with PBS, fixed and stained as described [46]. For each experiment, 30, 000 cells were analyzed in triplicate.

### Immunofluorescence analysis

Immunofluorescence optical sectioning microscopy was performed on an AxioObserver microscope equipped with a ApoTome.2 structured illumination unit using a 63x/1.4 NA oil immersion Plan-Apochromat objective (Carl Zeiss, Jena, Germany). DAPI, Alexa488 and Cy3 dyes were excited through dye-specific optical filters at 335–383 nm, 450–490 nm and 538–562 nm, respectively at 50% constant output of a HXP120 fluorescence excitation light source. DAPI, Alexa488 and Cy3 fluorescence was captured with dye-specific band pass filters at 420–470 nm, 500–550 nm and 570–640 nm, respectively. Beamsplitters at 395 nm, 495 nm, and 570 nm were used, respectively. There was no bleedthrough or cross-talk in the triple-fluorescence staining experiments using these filter combinations. Each dye was scanned independently in multitracking mode. Optical sections were acquired for each color channel at mid-nucleus postion after selection in the z-axis using an Axiocam 503 mono camera (Zeiss) at 1936 × 1460 pixels (72 nm per pixel). Acquisition settings (exposure times) were kept constant for all images and were as follows: 200 ms for DAPI, 1 s for Alexa 488 and 500 ms for Cy3. Optical sections with an axial resolution of 720 nm (DAPI), 790 nm (Alexa488) and 880 nm (Cy3) were achieved through structured illumination employing 3 phase images without any normalization, phase correction, Fourier filtering or deconvolution.

ZEN 2 pro software (Carl Zeiss) was used for image acquisition, display and export as TIF files. Micrographs were assembled from TIF files using Photoshop software.

We analyzed phospho-Rb to analyze the G1/S checkpoint control and p21 localization. DNA was stained with 2-(4-amidinophenyl)-6-indolecarbamidine dihydrochloride (DAPI) (Sigma-Aldrich, Taufkirchen, Germany). Polyclonal anti-pRb antibodies were used at a 1:250 dilution in 1% BSA (Sigma-Aldrich, Taufkirchen, Germany) in PBS, p21 antibodies at a 1:100 dilution in 1% in PBS.

### Statistical methods

All experiments were performed at least in triplicate. All treatments were compared with untreated control cells. Statistical analysis was performed with student's *t*-test and ANOVA to consider random effects as described [21]. *EC_50_* values were calculated from the cell proliferation experiments assuming the cell number of control cells at the latest time point as 100%.

The combination index was calculated using the following equation: c.i. = (Am)_50_/(As)_50_ + (Bm)_50_/(Bs)_50_, where (Am)_50_ is the concentration of drug A necessary to achieve a 50% inhibitory effect (*IC_50_*) in the combination, (As)_50_ is the concentration of the same drug that will produce the identical level of effect alone, (Bm)_50_ is the *IC_50_* of drug B in the combination and (Bs)_50_ is the *IC_50_* of drug B after single administration. Antagonism is indicated when c.i. > 1, c.i. = 1 indicates an additive effect and a c.i. < 1 indicates synergy [47].

## Abbreviations

Plk1: polo-like kinase 1
HDAC: histone deacetylase
HDACi: histone deacetylase inhibitor
SAHA: suberoylanilide hydroxamic acid
pRb: phospho-Rb
